# Fractional Flow Reserve-Guided Strategy in Acute Coronary Syndrome. A Systematic Review and Meta-Analysis

**DOI:** 10.5935/abc.20180170

**Published:** 2018-10

**Authors:** José Luís Martins, Vera Afreixo, José Santos, Lino Gonçalves

**Affiliations:** 1 Department of Cardiology, Baixo Vouga Hospital Center, Aveiro - Portugal; 2 CIDMA/IBIMED/Department of Mathematics, University of Aveiro, Aveiro - Portugal; 3 Department of Cardiology, Coimbra Universitary Hospital Center, Coimbra - Portugal

**Keywords:** Acute Coronary Syndrome/physiopathology, Percutaneous Coronary Intervention/methods, Coronary Angiography/methods, Fractional Flow Reserve Myocardial/physiology, Microvessels, Vascular Resistance, Reproducibility of Results

## Abstract

**Background:**

There are limited data on the prognosis of deferral of lesion treatment in patients with acute coronary syndrome (ACS) based on fractional flow reserve (FFR).

**Objectives:**

To provide a systematic review of the current evidence on the prognosis of deferred lesions in ACS patients compared with deferred lesions in non-ACS patients, on the basis of FFR.

**Methods:**

We searched Medline, EMBASE, and the Cochrane Library for studies published between January 2000 and September 2017 that compared prognosis of deferred revascularization of lesions on the basis of FFR in ACS patients compared with non-ACS patients. We conducted a pooled relative risk meta-analysis of four primary outcomes: mortality, cardiovascular (CV) mortality, myocardial infarction (MI) and target-vessel revascularization (TVR).

**Results:**

We identified 7 studies that included a total of 5,107 patients. A pooled meta-analysis showed no significant difference in mortality (relative risk [RR] = 1.44; 95% CI, 0.9-2.4), CV mortality (RR = 1.29; 95% CI = 0.4-4.3) and TVR (RR = 1.46; 95% CI = 0.9-2.3) after deferral of revascularization based on FFR between ACS and non-ACS patients. Such deferral was associated with significant additional risk of MI (RR = 1.83; 95% CI = 1.4-2.4) in ACS patients.

**Conclusion:**

The prognostic value of FFR in ACS setting is not as good as in stable patients. The results demonstrate an increased risk of MI but not of mortality, CV mortality, and TVR in ACS patients.

## Introduction

Fractional flow reserve is a well-validated, effective technique to determine the functional significance of intermediate coronary lesions; FFR-guided percutaneous coronary intervention (PCI) improves clinical outcomes in patients with stable coronary disease.^1^^-^^3^ Although robust data supports FFR use in stable coronary disease, its use in acute coronary syndrome (ACS) is less well investigated because maximal hyperemia is required to accurately measure FFR. In patients with ACS, microvascular changes may prevent vasodilatation thus affecting the validity of FFR.^1^^,^^4^^-^^6^ These changes appear to be vessel-dependent (culprit vs. non-culprit) and related to the type of infarction - ST-elevation myocardial infarction (STEMI) vs. non-ST-elevation myocardial infarction (NSTEMI).^7^ FFR values in the culprit vessel are recognized to be higher when measured during acute episodes than when measured after the microcirculation has had some time to recover. Higher FFR values are assumed to be caused by reduced levels of hyperemia in the culprit vessel due to embolization of thrombus and plaque, ischemic microvascular dysfunction and myocardial stunning. Hence, efficacy of the use of FFR in culprit artery disease remains uncertain.^8^^,^^9^

Multivessel coronary disease (MVD), observed in approximately 30-50% of patients presenting with STEMI and in 30-59% with NSTEMI, is associated with a poor prognosis.^10^^-^^12^ Complete revascularization of hemodynamically significant vessels identified in the hemodynamic laboratory early after acute event appears attractive: this approach provides the patient with a well-defined, definitive therapeutic plan. However, several studies suggest that a FFR-guided revascularization strategy in ACS reduces the rate of coronary revascularization without compromising short-term safety.^13^^-^^15^ However, the results of this approach are inconsistent in several studies involving patients with non-ACS.^13^^,^^14^

Therefore, the aims of this study are to provide a systematic review of the current evidence of the deferral of PCI based on FFR in ACS patients and compare it with that supporting this decision in non-ACS patients.

## Methods

### Data sources and searches

We systematically searched MEDLINE, EMBASE, and the Cochrane Library for relevant articles published between January 2000 and September 2017. Previous qualitative and systematic reviews, if available, were searched for additional studies. The query terms “Flow Fractional Reserve” OR “Acute Coronary Syndrome” were used in the search. References of the studies identified by the search strategy were reviewed for potentially relevant articles not identified by the above search. No language restrictions were enforced.

### Study selection

**Figure 1:**
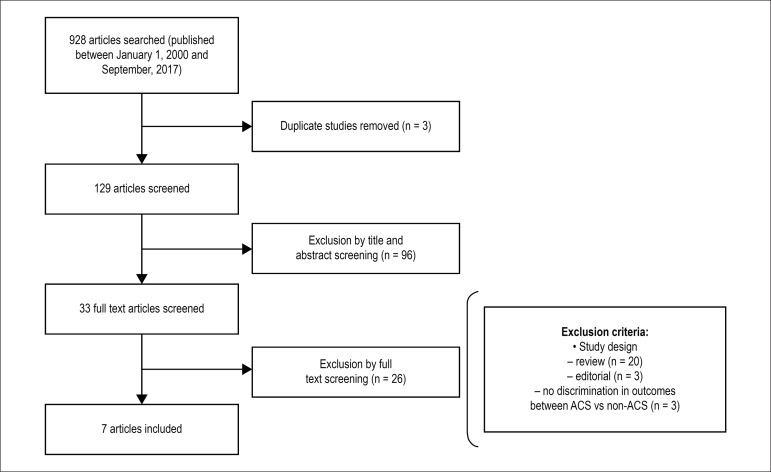
Flowchart of studies included in the meta-analysis.

The title/abstract of citations were first screened by 2 independent reviewers (JM and VA), and complete manuscripts were retrieved if considered potentially relevant. Additional studies were identified by reviewing the bibliographies of included studies and relevant reviews. Disagreements were resolved by consensus. The same reviewers independently appraised identified articles according to the following inclusion criteria: studies that compared clinical outcomes of lesions after PCI deferred based on FFR between ACS patients and non-ACS patients (Figure 1).

### Endpoints

The endpoints studied were: mortality, cardiovascular mortality, myocardial infarction (MI), and target vessel revascularization (TVR) during the follow-up period. TVR of the target vessel was defined as subsequent revascularization of the index vessel by either PCI or bypass grafting. In all trials, in the ACS group, distinction between culprit and non-culprit lesions was based on the operator's discretion, and hence subjective, similar to clinical practice.

### Statistical analysis

Continuous variables were expressed as means ± standard deviations or median (with interquartile range) values, and categorical variables were described as numbers and percentages. To calculate pooled effect estimates, we used the inverse variance assuming a fixed-effects model and the DerSimonian-Laird method assuming a random-effects model.^16^ Homogeneity among the studies was evaluated using Cochran's Q test and the I2 statistic (the values of 0.25, 0.50, and 0.75 indicated low, moderate, and high degrees of heterogeneity, respectively). Publication bias was evaluated using funnel plots. We performed a sensitivity analysis to evaluate the impact of each study on the results. MetaXL 2.0 (EpiGear International Pty Ltd, Wilston, Queensland, Australia) was used to calculate the pooled risk difference effect sizes (difference in occurrence risk between revascularization and conservative management groups).

## Results

### Study identification

The search strategy initially retrieved 129 citations. Of these, 96 articles were excluded after review of the title or abstract. After assessment or the studies for the selection criteria, we excluded an additional 26 studies. A total of 7 studies met criteria for the meta-analysis, involving 5,107 (3,540 non-ACS and 1,567 ACS) patients.

### Characteristics of included studies

Of the 7 studies included, 1 was a prospective study and 6 had an observational, retrospective in design (Table 1 and Table 2).

**Table 1:** Characteristics of included studies

Author	Year	Follow up	Study design	Total FU	Age (yrs)	Men	Non-ACS (n)	ACS (n)	STEMI (n)	NSTEMI/UA (n)	FFR value used to defer	Median time between Clinical presentation and FFR measurement	Multivessel disease	Adenosine administration	Exclusion criteria
Potvin JM et al ^9^	2006	11 ± 6 months	Retrospective cohort	201	62 ± 10	131	61	124	11	113	≥ 0.75	24 hours (range 2 to 144)	NR	intracoronary administration of adenosine (median dose 60 µg, range 30 to 300, for the left coronary artery and 30 µg, range 18 to 120, for the right coronary artery) and/or nitroprusside (median dose 250 µg, range 100 to 1,000, for the left and right coronary arteries). Intracoronary adenosine was used in 135 cases, intracoronary nitroprusside in 14 cases, and adenosine andnitroprusside in 52 cases	Patients within 24 hours of acute STEMI were excluded
Fischer J et al ^8^	2006	12 months	Retrospective cohort	111	ACS → 58 ± 14Non-ACS → 63 ± 10	72	76	35	11	24	≥ 0.75	Recent(within 7 days) ST segment elevation MI treated with lytic Therapy	ACS → 9Non-ACS → 9	intracoronary adenosine (30 µg bolus in the right coronary artery or 40-60 µg bolus in the left coronary artery	NR
Sels et al ^24^	2011	2 years	Prospective cohort	1005	ACS → 64.8 ± 10.7Non-ACS → 64.3 ± 10	744	677	328	0	328	≥ 0.80	NR	NR	Intravenous adenosine, administered at a rate of 140 µg/kg/min through a central vein.	Exclusion criteria were left main disease, previous CABG, and STEMI < 5 days before, because the use of FFR is not validated in recent STEMI. Patients admitted for UA and NSTEMI with positive troponin but total creatine kinase < 1,000 U/l could be included
Mehta et al ^25^	2015	3.4 ± 1.6 years	Retrospective cohort	674	ACS → 63.8 ± 11.9Non-ACS → 65.3 ± 10.2	380	340	334	7	327	> 0.80	NR	ACS → 221Non-ACS → 209	Predominant use of intracoronary adenosine with similar maximum doses for both groups (120 µg)	NR
Hakeem A et al ^34^	2016	3,4 ± 1,6 anos	Retrospective cohort	576	ACS → 66.6 ± 8Non-ACS → 64.7 ± 8.7	554	370	206	0	206	> 0.75	NR	ACS → 135Non-ACS → 216	Intravenous (140 mg/kg/min) or intracoronary (at least 60 mg) adenosine. The median dose of intracoronary adenosine in our cohort was 130 mg	NR
Van Belle et al ^38^	2017	1 year	Retrospective cohort	958	ACS → 66 ± 11.2Non-ACS → 66.4 ± 10	693	721	237	-	-	> 0.75 e > 0.80	NR	NR	NR	NR
Lee JM et al ^37^	2017	722 days	Retrospective cohort	1596	ACS → 62.0 ± 11.1Non-ACS → 62.4 ± 9.4	1112	1295	301	0	301	> 0.80	NR	NR	Hyperemia was induced with an intracoronary bolus administration (80 µg in left coronary artery, 40 µg in right coronary artery), intracoronary (240 µg/min) or, iv continuous infusion (140 µg/Kg/min) of adenosine.	NR

FU: Follow-up; yrs: years; ACS: acute coronary syndrome; STEMI: ST segment elevation myocardial infarction; NSTEMI: non- ST segment elevation myocardial infarction; UA: unstable angina; FFR: fractional flow reserve; PCI: Percutaneous coronary intervention; MI: Myocardial Infarction; TVR: target vessel revascularization; CABG: Coronary artery bypass grafting; NR: not reported.

**Table 2:** Clinical outcomes of ACS and non-ACS patients with deferred lesion treatment based on fractional flow reserve

Author	Year	Patients[FFR > cutoff] *	Mortality	CV Mortality	Myocardial infarction	Target lesion revascularization	Target vessel revascularization
Potvin JM et al ^9^	2006	ACS → 124Non-ACS → 61	NR	ACS → 0Non-ACS → 1	ACS → 2Non-ACS → 1	NR	ACS → 11Non-ACS → 7
Fischer J. et al ^8^	2006	ACS → 35Non-ACS → 76	ACS → 3Non-ACS → 5	ACS → 2Non-ACS → 1	ACS → 1Non-ACS → 1	NR	ACS → 6Non-ACS → 7
Sels et al ^24^	2011	NR**	ACS → 12Non-ACS → 20	NR	Non-ACS → 36Non-ACS → 44	NR	ACS → 45Non-ACS → 72
Mehta et al ^25^	2015	ACS → 334Non-ACS → 340	NR	ACS → 23 Non-ACS → 8	ACS → 47Non-ACS → 26	ACS → 78Non-ACS → 66	NR
Hakeem A et al ^34^	2016	ACS → 206Non-ACS → 370	NR	ACS → 9Non-ACS → 30	ACS → 16Non-ACS → 11	ACS → 36Non-ACS → 29	ACS → 15 Non-ACS → 14
Van Belle et al ^38^	2017	ACS → 237Non-ACS → 721	ACS → 10Non-ACS → 17	NR	ACS → 3Non-ACS → 7	NR	NR***ACS → 9;***Non-ACS → 42]
Lee JM et al ^37^	2017	ACS → 301Non-ACS 1295	NR	ACS → 3Non-ACS → 5	ACS → 2Non-ACS → 4		ACS → 8Non-ACS → 10

ACS: acute coronary syndrome; CV: cardiovascular; NR: not reported;

* Cut-off values varied from 0.75 to 0.80 among the studies;

** Sels et al.^24^ evaluated whether there is a difference in benefit of fractional flow reserve (FFR) guidance for percutaneous coronary intervention (PCI) in multivessel coronary disease in patients with acute coronary syndrome (ACS) vs. non-ACS without discriminating those patients with FFR > 0.80;

*** Target-vessel revascularization was not specified.

### Quantitative synthesis of outcomes

**Figure 2:**
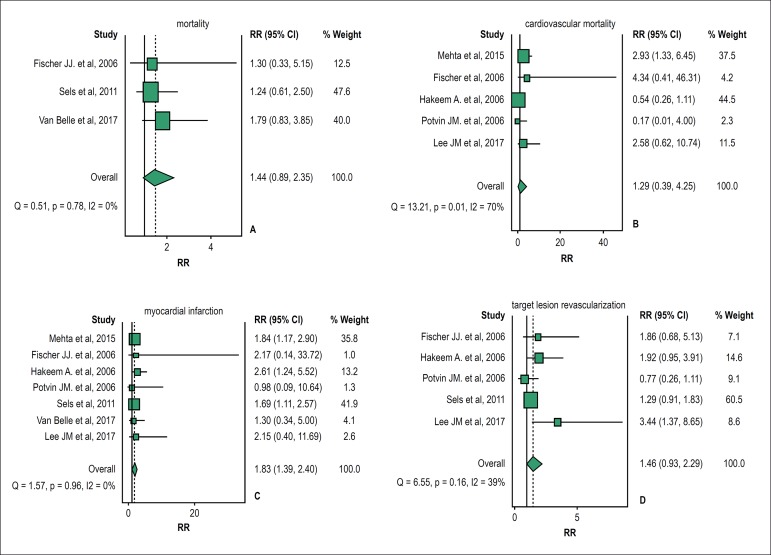
Forest plots of the pooled risk ratio of the outcomes. (A) mortality, (B) cardiovascular mortality; (C) myocardial infarction; (D) target-vessel revascularization. Size of data markers reflects the relative weight of the study. CI indicates confidence interval.

**Mortality:** We included 3 studies, a total of 2,074 patients, in the pooled analysis. The forest plot (Figure 2) describes the weighted meta-analysis for relative risk (RR) of mortality in ACS patients in comparison with non-ACS patients when revascularization decisions were based on FFR. Pooled analysis showed negligible heterogeneity among the studies (I2 = 0%; p = 0.78) and the ACS and non-ACS patients did not differ significantly; their pooled RR was 1.44 (95% CI = 0.89-2.35). Exclusion of any single study did not significantly alter the overall combined result.

**Cardiovascular mortality:** We included 5 studies, a total of 3,144 patients, in the pooled analysis. The forest plot (Figure 2) describes the weighted meta-analysis for mortality risk of basing revascularization decisions on FFR. Pooled analysis showed significant heterogeneity among the studies (I2 = 70%; p = 0.01) and the ACS and non-ACS patients did not differ significantly; their pooled RR was 1.29 (95% CI = 0.39-4.25). Exclusion of any single study did not significantly alter the overall combined result.

**Myocardial Infarction:** 7 studies were included, a total of 5,107 patients, in the pooled analysis. Deferring lesions based on FFR was associated with a significant additional risk of MI (RR = 1.83; 95% CI = 1.39-2.40) in ACS patients versus non-ACS patients. Figure 2 describes the weighted meta-analysis of MI. The pooled analysis showed negligible heterogeneity among the studies (I2 = 0%; p = 0.96).

**Target-vessel revascularization:** We included 5 studies, a total of 3,475 patients, in the pooled analysis. The forest plot (Figure 2) describes the weighted meta-analysis of TVR in patients when revascularization decisions were based on FFR. Pooled analysis showed negligible heterogeneity among the studies (I2 = 39%; p = 0.16). ACS and non-ACS patients did not differ significantly in RR of TVR; their pooled RR was 1.46 (95% CI = 0.93-2.29).

### Study Bias

Visual inspection of the funnel plots for the outcomes did not reveal any asymmetry among the studies. Further, the Begg rank correlation test was not statistically significant.

## Discussion

This report provides a systematic review and a meta-analysis comparing the strategy in patients in whom lesion treatment was deferred based on FFR, and no revascularization was undertaken in ACS patients to that in non-ACS patients. FFR-guided revascularization in ACS patients appears to be as safe as in non-ACS patients.^2^^,^^17^^-^^18^ Briasoulis et al.,^15^ in a meta-analysis, evaluated FFR-guided management in NSTEMI patients, where a modest reduction in incidence of MI was noted, with no significant differences in incidence of major adverse cardiac events (MACE), death or all-cause mortality, and target-vessel revascularization between the FFR guided approach in comparison with coronary angiography-guided approach.^15^

Four important pathophysiological considerations need to be considered when comparing the FFR results in ACS patients to those of non-ACS patients:

1. **Microvascular dysfunction:** The timing of FFR measurement in the ACS patient is an important issue. As described above, immediately after MI, the initial, temporary microvascular injury caused by the inflammatory environment may artificially elevate the initial FFR measurements. Antithrombotic therapy, administered for 3 to 4 days to stabilize the plaque, may reduce microvascular dysfunction, and FFR may then reflect the true hemodynamic situation. This approach of waiting > 5 days to measure FFR in ACS patients was suggested by the European Society of Cardiology guidelines.^19^^-^^21^ However, most referral centers that study FFR in ACS perform early invasive evaluation of ACS patients, within 48 h of presentation, a practice that could lead to artificially higher FFR values.^19^^,^^22^^-^^27^^,^^34^^,^^37^^,^^38^
2. **Plaque instability:** At least two-thirds of lesions arising from vessels with < 50% stenosis are responsible for unstable syndromes involving plaque instability, assuming that these vessels previously had normal flow. A non-flow-limiting culprit lesion may be "anatomically significant" but "physiologically nonsignificant", and because FFR is not intended to evaluate plaque characteristics, care must be taken in the use of FFR in vessels with unstable characteristics but normal flow.^28^^,^^29^
3. **Myocardial mass involved:** The mass of viable myocardium being perfused by the artery in question is relevant pathophysiologically to the interpretation of FFR results in ACS patients. The FFR value is inversely proportional to the ejection fraction: hence, a lower ejection fraction, which implies a large area of infarction with less viable myocardium, could produce a higher FFR reading for the same degree of stenosis.^14^^,^^30^
4. **Presentation type of ACS:** Because ACS describe a range of myocardial ischemic states with distinct clinical and pathophysiological characteristics, the use of FFR should be differentiated by type of ACS. DANAMI3-PRIMULTI and COMPARE ACUTE were the only studies that evaluated the risk of events following FFR-guided PCI in patients with STEMI and MVD.^31^^,^^32^ Of these, only COMPARE ACUTE reported the rate of events at follow-up in patients whose PCI was deferred based on FFR; patients who did not undergo additional revascularization had a similar event rate to those who were revascularized based on positive (elevated) FFR. On the other hand, FAME, which included 328 patients with ACS out of a total of 1,005 patients with MVD, reported similar rates of mortality, MI, or revascularization in non-ST-segment elevation acute coronary syndrome (NSTE-ACS) patients who had PCI deferred based on an FFR cutoff value > 0.80 compared to non-ACS patients.^24^ However, the FAME study did not define the exact time of FFR measurement nor the lesions assessed (culprit *vs*. non-culprit). Furthermore, the event rate in patients with deferred PCI based on FFR was not reported. In addition, the FAMOUS-NSTEMI trial compared a FFR-guided versus an angiography-only approach in NSTEMI and MVD patients; the rate of major adverse cardiac events (defined as cardiac mortality or hospitalization for MI or heart failure) was 7.5% in patients with deferred PCI based on FFR and 0% in those deferred PCI based on angiography.^13^

The aim of this analysis was not to evaluate FFR-guided decisions per-lesion level, but rather to focus on the relevance of FFR-guided decision per-patient level, considering that patients with ACS frequently have more than 1 lesion suitable for revascularization and the identification of the culprit lesion is not always straightforward. Undoubtedly, patients with MVD have worse outcomes than patients who present with single vessel disease. The natural history of patients who are revascularized in an acute setting is known to differ from those who are revascularized in a stable setting.^33^ For example, the probability of malignant dysrhythmias is significantly more common in acute patients and is an important cause of mortality.^33^

This systematic review and meta-analysis summarizes all published studies that assessed and compared clinical outcomes in which revascularization decisions were based on FFR in ACS versus non-ACS setting. Among the clinical endpoints evaluated, only the RR of MI was significantly higher in patients with ACS.

The higher risk of subsequent MI found in this study and by several authors is explained by the different pathophysiology of ACS versus stable coronary disease.^34^^-^^36^ Hakeem et al. compared the outcomes in NSTEMI patients who did not undergo PCI of any lesion on the basis of FFR to those in a similar group of non-ACS patients. After an average 3.4-years follow-up, using propensity score matching, the MI and TVR rates were higher in NSTEMI patients than in non-ACS patients (25% vs. 12%, respectively; p < 0.0001).^34^ Similar results were reported recently by Lee et al. in non-ACS patients.^37^

**Figure 3:**
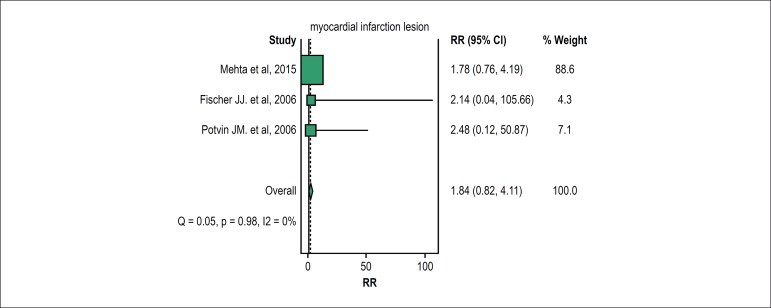
Forest plot of the pooled risk ratio for myocardial infarction injury. Size of data markers reflects the relative weight of the study. CI indicates confidence interval.

When MI injury (defined as any MI attributable to a deferred revascularization based on the index FFR) was specifically evaluated, deferring treatment of lesions based on FFR did not differ significantly in the RR of MI injury between ACS and non-ACS patients [RR 1.84 (95% CI = 0.82-4.11); (I2 = 0%; p = 0.98)] (Figure 3).

If on the one hand, Briasoulis et al.^15^ showed that a FFR-guided strategy in ACS seems to be associated with a better prognosis compared to an angiography strategy, the primary finding of our study was that deferring the treatment of lesions was associated with an increased risk of MI in ACS patients compared to non-ACS patients, represented by the RRs of the target-vessel revascularization or MI lesion.^15^ In addition, mortality and CV mortality did not differ between ACS and non-ACS patients.

Our results are consistent with the recently published study by Van Belle et al.,^38^^,^^39^ who compared the impact differing the management of intermediate lesions, based on FFR, on the prognosis of ACS *vs*. non-ACS patients from two important registries, R3F and POST-iT. They concluded that revascularization decisions based on FFR for differing treatment of lesions were safe in ACS patients.^38^^-^^40^

Some authors have questioned whether we should be less permissive and adopt a different cut-off value for FFR in unstable vessels. Hakeem et al.,^34^ recently determined that the best FFR cut-off value for predicting MI or TVR was > 0.80 in patients with stable coronary artery disease, supporting current practice. However, in NSTE-ACS patients, the best cutoff value was >0.84. However, some limitations suggested by some authors deserve consideration in interpreting their results. For example, it is unclear why mortality, the most important outcome, was not included in the composite endpoint in this study. In addition, medical therapy was not optimal for the patients, 14% of patients did not receive statin, and approximately two-thirds did not receive dual antiplatelet therapy. Moreover, several technical issues might explain the higher FFR cut-off values reported in these studies.^34^^,^^41^^-^^43^

Despite most of the studies included did not report clinical outcomes by type of lesions (culprit or non-culprit) lesions, available evidence suggests, as previously mentioned, that in patients with ACS, microvascular dysfunction may be less marked, and the ability to achieve maximal hyperemia is sufficient to maintain the diagnostic use of FFR, both in culprit and non-culprit vessels.^44^

Besides that, due to the great heterogeneity of inclusion criteria, follow-up period and the vessel assessed by FFR, results and conclusion of the current study should be the interpreted with caution.

### Limitations

The conclusions drawn from this meta-analysis are subject to the limitations and differences of the original studies included in the analysis. First, our meta-analysis included both randomized clinical trials and (mostly) observational studies. The conclusions of this study may be somewhat limited due to biases inherent in the observational studies, including design, selection, and treatment bias. Another possible limitation is the potential publication bias because the results only included short-term mortality.

## Conclusion

The prognostic value of FFR in ACS setting is not as good as in stable patients. More homogeneous studies with larger populations of patients are necessary to reach definitive and robust conclusions. Careful definition and interpretation of the clinical results is important when analysis of FFR is not performed in patient-level but in vessel-level only.

## References

[1] De Bruyne B, Sarma J (2008). Fractional flow reserve: a review: invasive
imaging. Heart.

[2] Pijls NH, van Schaardenburgh P, Manoharan G, Boersma E, Bech JW, van't Veer M (2007). Percutaneous coronary intervention of functionally nonsignificant
stenosis: 5-year follow-up of the DEFER Study. J Am Coll Cardiol.

[3] Zimmermann FM, Ferrara A, Johnson NP, van Nunen LX, Escaned J, Albertsson P (2015). Deferral vs. performance of percutaneous coronary intervention of
functionally non-significant coronary stenosis: 15-year follow-up of the
DEFER trial. Eur Heart J.

[4] Tamita K, Akasaka T, Takagi T, Yamamuro A, Yamabe K, Katayama M (2002). Effects of microvascular dysfunction on myocardial fractional
flow reserve after percutaneous coronary intervention in patients with acute
myocardial infarction. Catheter Cardiovasc Interv.

[5] Tani S, Watanabe I, Kobari C, Matsumoto M, Miyazawa T, Iwamoto Y (2004). Mismatch between results of myocardial fractional flow reserve
measurements and myocardial perfusion SPECT for identification of the
severity of ischaemia. Jpn Heart J.

[6] Yong AS, Fearon WF (2013). Coronary microvascular dysfunction after ST-segment elevation
myocardial infarction: local or global phenomenon?. Circ Cardiovasc Interv.

[7] Kumar A, Cannon CP (2009). Acute coronary syndromes: diagnosis and management, Part
I. Mayo Clin Proc.

[8] Fischer JJ, Wang XQ, Samady H, Sarembock IJ, Powers ER, Gimple LW (2006). Outcome of patients with acute coronary syndromes and moderate
coronary lesions undergoing deferral of revascularisation based on
fractional flow reserve assessment. Catheter Cardiovasc Interv.

[9] Potvin JM, Rodes-Cabau J, Bertrand OF, Gleeton O, Nguyen CN, Barbeau G (2006). Usefulness of fractional flow reserve measurements to defer
revascularisation in patients with stable or unstable angina pectoris, non
ST-elevation and ST-elevation myocardial infarction, or atypical chest
pain. Am J Cardiol.

[10] Lekston A, Tajstra M, Gasior M, Gierlotka M, Pres D, Hudzik B (2011). Impact of multivessel coronary disease on one-year clinical
outcomes and five-year mortality in patients with ST-elevation myocardial
infarc- tion undergoing percutaneous coronary intervention. Kardiol Pol.

[11] Dziewierz A, Siudak Z, Rakowski T, Zasada W, Dubiel JS, Dudek D (2010). Impact of multivessel coronary artery disease and
noninfarct-related artery revascularization on outcome of patients with
ST-elevation myocardial infarction transferred for primary percutaneous
coronary intervention (from the EUROTRANSFER Registry). Am J Cardiol.

[12] Dellavalle A, De Servi S, Repetto S, Chierchia S, Repetto A, Vado A (2000). Coronary angioplasty in patients with unstable angina: clinical,
electrocardiographic and angiographic predictors of in-hospital
outcome. R.OS.A.I. Study Group. Ital Heart J.

[13] Layland J, Carrick D, McEntegart M, Ahmed N, Payne A, McClure J (2013). Vasodilatory capacity of the coronary microcirculation is
preserved in selected patients with non-ST-segment-elevation myocardial
infarction. Catheter Cardiovasc Interv.

[14] Henningam B, Layland J, Fearon WF, Oldroyd KG (2014). Fractional flow reserve and the index of microvascular resistance
in patients with acute coronary syndromes. EuroIntervention.

[15] Briasoulis A, Palla M, Mostafa A, Afonso L, Grines C (2015). Fractional flow-guided management in patients with acute coronary
syndromes: a systematic review and meta-analysis. Int J Cardiol.

[16] DerSimonian R, Laird N (1986). Meta-analysis in clinical trials. Control Clin Trials.

[17] Echavarría-Pinto M, van de Hoef TP, Serruys PW, Piek JJ, Escaned J. (2014). Facing the complexity of ischaemic heart disease with
intracoronary pressure and flow measurements: beyond fractional flow reserve
interrogation of the coronary circulation. Curr Opin Cardiol.

[18] onino PA, De Bruyne B, Pijls NH, Siebert U, Ikeno F, van't Veer M, FAME Study Investigators (2009). Fractional flow reserve versus angiography for guiding
percutaneous coronary intervention. N Engl J Med.

[19] Depta JP, Patel JS, Novak E, Masrani SK, Raymer D, Facey G (2014). Outcomes of coronary stenoses deferred revascularization for
borderline versus nonborderline fractional flow reserve
values. Am J Cardiol.

[20] Layland J, Oldroyd KG, Curzen N, Sood A, Balachandran K, Das R, FAMOUS-NSTEMI investigators (2015). Fractional flow reserve vs. angiography in guiding management to
optimize outcomes in non-ST-segment elevation myocardial infarction: the
British Heart Foundation FAMOUS-NSTEMI randomized trial. Eur Heart J.

[21] Kolh P, Windecker S (2014). ESC/EACTS myocardial revascularization guidelines
2014. Eur Heart J.

[22] Niccoli G, Indolfi C, Davies JE (2017). Evaluation of intermediate coronary stenoses in acute coronary
syndromes using pressure guidewire. Open Heart.

[23] Lopez-Palop R, Carrillo P, Torres F, Lozano I, Frutos A, Avanzas P (2012). Results of fractional flow reserve measurement to evaluate
nonculprit coronary artery stenoses in patients with acute coronary
syndrome. Rev Esp Cardiol (Engl Ed).

[24] Sels JW, Tonino PA, Siebert U, Fearon WF, Van't Veer M, De Bruyne B (2011). Fractional flow reserve in unstable angina and non-ST-segment
elevation myocardial infarction experience from the FAME (Fractional flow
reserve versus angiography for Multivessel evaluation) study. JACC Cardiovasc Interv.

[25] Masrani Mehta S, Depta JP, Novak E, Patel JS, Patel Y, Raymer D (2015). Association of lower fractional flow reserve values with higher
risk of adverse cardiac events for lesions deferred revascularization among
patients with acute coronary syndrome. J Am Heart Assoc.

[26] Picchi A, AntonioMaria Leone AM, Zilio F, Enrico Cerrato E, D'Ascenzo F, Fineschi M (2017). Outcome of coronary lesions with deferred revascularization due
to negative fractional flow reserve in subjects with acute coronary
syndrome. Int J Cardiol.

[27] Leesar MA, Abdul-Baki T, Akkus NI, Sharma A, Kannan T, Bolli R (2003). Use of fractional flow reserve versus stress perfusion
scintigraphy after unstable angina. Effect on duration of hospitalization,
cost, procedural characteristics, and clinical outcome. J Am Coll Cardiol.

[28] Camici PG, Crea F (2007). Coronary microvascular dysfunction. N Engl J Med.

[29] Lerman A, Zeiher AM (2005). Endothelial function: cardiac events. Circulation.

[30] De Bruyne B, Pijls NH, Bartunek J, Kulecki K, Bech JW, De Winter H (2001). Fractional flow reserve in patients with prior myocardial
infarction.. Circulation.

[31] Engstrøm T, Kelbæk H, Helqvist S, Høfsten DE, Kløvgaard L, Holmvang L, DANAMI-3-PRIMULTI Investigators (2015). Complete revascularization versus treatment of the culprit lesion
only in patients with STsegment elevation myocardial infarction and
multivessel disease (DANAMI-3-PRIMULTI): an open-label, randomised
controlled trial. Lancet.

[32] Smits PC, Abdel-Wahab M, Neumann FJ, Boxma-de Klerk BM, Lunde K, Schotborgh CE, Compare-acute investigators (2017). fractional flow reserve-guided multivessel angioplasty in
myocardial infarction. N Engl J Med.

[33] Alcock RF, Yong AS, Ng AC, Chow V, Cheruvu C, Aliprandi-Costa B (2013). Acute coronary syndrome and stable coronary artery disease: are
they so different? Long-term outcomes in a contemporary PCI
cohort. Int J Cardiol.

[34] Hakeem A, Edupuganti MM, Almomani A, Pothineni NV, Payne J, Abualsuod AM (2016). Long-term prognosis of deferred acute coronary syndrome lesions
based on nonischemic fractional flow reserve. Am Coll Cardiol.

[35] Morrow DA (2010). Cardiovascular risk prediction in patients with stable and
unstable coronary heart disease. Circulation.

[36] Mahaffey KW, Wojdyla DM, Pieper KS, Tricoci P, Alexander JH, Lincoff AM (2014). Comparison of clinical trial outcome patterns in patients
following acute coronary syndromes and in patients with chronic stable
atherosclerosis. Clin Cardiol.

[37] Lee JM, Choi KH, Koo BK, Shin ES, Nam CW, Doh JH (2017). Prognosis of deferred non-culprit lesions according to fractional
flow reserve in patients with acute coronary syndrome. Eurointervention.

[38] Van Belle E, Baptista SB, Raposo L, Henderson J, Rioufol G, Santos L, PRIME-FFR Study Group (2017). Impact of Routine Fractional Flow Reserve on Management Decision
and 1-Year Clinical Outcome of Patients With Acute Coronary Syndromes:
PRIME-FFR (Insights From the POST-IT [Portuguese Study on the Evaluation of
FFR-Guided Treatment of Coronary Disease] and R3F [French FFR Registry]
Integrated Multicenter Registries - Implementation of FFR [Fractional Flow
Reserve] in Routine Practice). Circ Cardiovasc Interv.

[39] Van Belle E, Rioufol G, Pouillot C, Cuisset T, Bougrini K, Teiger E, Investigators of the Registre Français de la FFR-R3F (2014). Outcome impact of coronary revascularization strategy
reclassification with fractional flow reserve at time of diagnostic
angiography: insights from a large French multicenter fractional flow
reserve registry. Circulation.

[40] Baptista SB, Raposo L, Santos L, Ramos R, Calé R, Jorge E (2016). Impact of routine fractional flow reserve evaluation during
coronary angiography on management strategy and clinical outcome: one-year
results of the prospective POST-IT multicenter registry. Circ Cardiovasc Interv.

[41] Fearon WF, De Bruyne B, Pijls NHJ (2016). Fractional flow reserve in acute coronary
syndromes. J Am Coll Cardiol.

[42] Rodés-Cabau J, Gutiérrez M, Courtis J, Larose E, Déry JP, Côté M (2011). Importance of diffuse atherosclerosis in the functional
evaluation of coronary stenosis in the proximal-mid segment of a coronary
artery by myocardial fractional flow reserve measurements. Am J Cardiol.

[43] Adjedj J, Toth GG, Johnson NP, Pellicano M, Ferrara A, Floré V (2015). Intracoronary adenosine: dose-response relationship with
hyperemia. JACC Cardiovasc Interv.

[44] Esen AM, Acar G, Esen O, Emiroglu Y, Akcakoyun M, Pala S (2010). The prognostic value of combined fractional flow reserve and TIMI
frame count measurements in patients with stable angina pectoris and acute
coronary syndrome. J Interv Cardiol.

